# Differentially expressed microRNAs in human depression: a systematic review of case-control and longitudinal studies

**DOI:** 10.1186/s12888-025-07054-1

**Published:** 2025-07-01

**Authors:** Yangyang He, Sanne Houtenbos, Pia-Maria Wippert

**Affiliations:** 1 Medical Sociology and Psychobiology, University of Potsdam, Am Neuen Palais 10, 14469 Potsdam, Germany; 2 Faculty of Health Sciences Brandenburg, Joint Faculty of the University of Potsdam, the Brandenburg Medical School Theodor Fontane and the Brandenburg University of Technology Cottbus– Senftenberg, Am Mühlenberg 9, 14476 Potsdam, Germany

**Keywords:** Mental disorder, Depressive disorder, Stress, Non-coding RNA

## Abstract

**Background:**

Depression is a complex psychiatric condition characterized by a significant disturbance in an individual’s cognition, emotional regulation, and/or behavior. Previous studies have investigated potential cellular and molecular mechanisms for depression, including microRNA (miRNA), which are small, non-coding RNAs that affect several biological processes involved in the development of depression. This systematic review aims to synthesize current evidence on altered miRNA expression in depressed patients, thus in an effort to understand the intricate interactions between depression and miRNAs for upcoming diagnoses and therapies.

**Methods:**

A systematic literature search was conducted in PubMed and Web of Science databases in November 2023. Studies were eligible if they (1) involved human depression studies, (2) investigated miRNA alterations, and (3) conducted case-control or longitudinal studies. After assessing the quality of studies with the NIH Quality Assessment Tool, the roles of altered miRNAs in depressed patients were synthesized.

**Results:**

A total of 1452 studies were screened, and 37 studies were finally included (26 case-control and 11 longitudinal studies; *n* = 2909 patients), in which 48 different miRNA alterations among depressed patients were observed. The seven miRNAs that were most frequently studied and consistently exhibited altered expression across the included studies were miR-146a-5p, miR-132-3p, miR-124-3p, miR-16-5p, miR-155-5p, miR-135a-5p, and miR-451a, which mostly play a role in the release of molecules involved in neurobiological processes.

**Conclusions:**

This systematic review illustrated the involvement of various miRNAs in the pathophysiology of depression, and identified miRNAs as potential diagnostic or therapeutic markers. These findings may contribute to the current understanding of miRNA-based biomarkers and new treatments for depression.

**Clinical trial number:**

Not applicable.

**Supplementary Information:**

The online version contains supplementary material available at 10.1186/s12888-025-07054-1.

## Introduction

Depression is a complex mental health disorder with significant psychobiological and psychosocial components, and is considered one of the main contributors to the Global Burden of Disease [1]. In Germany, the prevalence of depression increased strongly from 2009 (12.5%) to 2017 (15.7%) [2]. Furthermore, as a consequence of COVID-19 in 2020, the increasing SARS-CoV-2 infection rates and decreasing human mobility are associated with the increased prevalence of depression and anxiety disorders [3]. Traditionally, previous studies have revealed the potential pathophysiology involved in depression, such as structural changes in the brain [4], and inflammation imbalances [5]. Recently, explorations have suggested that novel pathways for the development of depression on the cellular biological level would be valuable for upcoming research and prevention strategies [6].

The pathophysiology of depression contains many common cellular mechanisms, for instance, neurotransmitter dysregulation [7], neuroinflammation [8], hypothalamic-pituitary-adrenal (HPA) axis dysregulation [9], epigenetic modifications [10], and oxidative stress [11]. Epigenetic modifications refer to the processes of nearby chromatin structures controlling specific gene activity, characterized by heritable changes in gene expression without changes in DNA sequence, which is essential for developing the nervous system [12]. The main epigenetic modification mechanisms include DNA methylation, histone modifications, and non-coding RNAs [13]. Among them, microRNAs (miRNAs), a class of small non-coding RNAs, have gained the attention of many researchers due to their functional properties. MiRNAs’ tiny molecules exert their regulatory functions by binding to the 3’ untranslated region of their target messenger RNAs, leading to their degradation or translational repression, and consequently inhibiting the synthesis of related proteins [14]. Increasing research has demonstrated the roles of altered miRNAs in the pathogenesis of different diseases, such as neurodegenerative diseases [15]. Furthermore, the altered miRNA expression profiles have recently served as potential diagnostic biomarkers for several disorders due to their disease-specific and tissue-specific expression [16]. Indeed, the regulatory effects of miRNAs are involved in various pathophysiological processes related to depression, including synaptic plasticity [17], neuroinflammation [18], and oxidative stress [19]. For example, in response to stress, miRNAs regulate neuroinflammation by influencing cytokine activation pathways and impacting the development of synapses [18]. Thus, miRNAs were regarded as a vital element in depression [20].

Considering the established interaction between depression and miRNAs, this systematic review aims to comprehensively collect and review the available research focused on altered miRNA expression in depression and their corresponding role. The specific miRNAs implicated in depression pathophysiology will be explored, which will provide more understanding of depression at a cellular level, paving the way for effective diagnosis and treatment.

## Methods

A literature search was performed in PubMed and Web of Science databases on 14 November 2023. Keywords for Searching in PubMed and Web of Science included “depression”, “depressive disorder”, and “miRNA”.

Search strategy in PubMed was used as: ((“depressed“[All Fields] OR “depression“[MeSH Terms] OR “depression“[All Fields] OR “depressions“[All Fields] OR “depressive disorder“[MeSH Terms] OR (“depressive“[All Fields] AND “disorder“[All Fields]) OR “depressive disorder“[All Fields] OR “depressivity“[All Fields] OR “depressive“[All Fields] OR “depressively“[All Fields] OR “depressiveness“[All Fields] OR “depressives“[All Fields])) AND (“micrornas“[MeSH Terms] OR “micrornas“[All Fields] OR “mirna“[All Fields] OR “mirnas“[All Fields])) NOT (“review“[Publication Type] OR “review literature as topic“[MeSH Terms] OR “review“[All Fields])

Search strategy in Web of Science was used as: (“depression” OR “depressive disorder”) AND “miRNA” NOT “review”.

EndNote 20 software (Thomson Reuters, Philadelphia, USA) was used to extract and manage the references. In addition, the references of all final eligible studies were also manually checked for potential papers. Although this systematic review was not registered on PROSPERO, it followed the guidelines of the Preferred Reporting Items for Systematic Reviews and Meta-Analyses (PRISMA) 2020 statement [21].

### Eligibility criteria

Inclusion criteria: original studies (case-control and cohort studies, RCTs), with full-texts in English, that investigate alterations in miRNAs (only mature miRNA) in adults (with and without depression) and include different tissues or bodily fluids from humans. To summarize miRNAs with more specific alterations, only consistent results validated by qPCR were accepted, along with those where miRNA expression level assays using microarray and sequencing.

Exclusion criteria: Studies that did not report differentially expressed miRNAs related to depression; non-primary research articles as well as technical notes, conference abstracts, reviews, news articles, letters, commentaries, and editorials; papers that derived miRNA expression data from previous research or online databases; articles solely focused on non-relevant outcomes for the current study; studies involving patients currently receiving treatment with medication; studies of patients with comorbid conditions, including hypertension, diabetes mellitus, psychosis, stroke, substance use disorders (drug or alcohol abuse), and infectious diseases; animal or cell model studies; and articles for which the full-text was not available were excluded.

### Literature selection

After excluding the duplicated references from all detected studies in the databases, two independent reviewers double-screened titles and abstracts according to the eligibility criteria. When the abstract met the eligibility criteria, the full text was obtained for further review. Then, the same procedure was conducted using the full text to include the final eligible studies. In case of disagreements, both reviewers discussed the inclusion of specific papers to reach a consensus.

### Data extraction and synthesis

For all final included manuscripts, the characteristics of each study (author, year, research design, sample size, etc.) were collected. The significantly altered (up- and down-regulated) miRNAs were reported based on statistical criteria set by individual studies, and the direction of altered miRNAs was reported as in the depression group compared with the control group. Then, for those recurrent miRNAs across different studies (occurrences ≥ 3), the determined roles of each miRNA from the included paper were synthesized. Considering the differences in publication time, miRNAs in the included studies were named based on different versions of naming convention rules. In order to avoid confusion regarding differentiated miRNA names in data integration, all miRNAs were named according to the consistent naming rules of miRBase V22.1.

### Study quality assessment

The NIH Quality Assessment Tool was used to assess the quality of each study. This tool, including 12 dichotomous items, evaluates the risk of bias in case-control studies based on various key domains, such as study population, sample size, inclusion/exclusion criteria, statistical analysis, etc. Each domain was rated as “yes,” “no,” “cannot determine,” “not applicable,” or “not reported.” Studies are assigned a score of “1” if the specific criterion is adequately addressed, for a total possible score of 12 (high quality). This assessment tool does not have a predefined cutoff score; two independent reviewers performed the study quality assessment based on the specific objectives and requirements of this systematic review; if there were any disagreements, they were discussed by both reviewers to reach consensus or consultation with a third reviewer when necessary.

## Results

### Literature screening and quality assessment

**Fig. 1:**
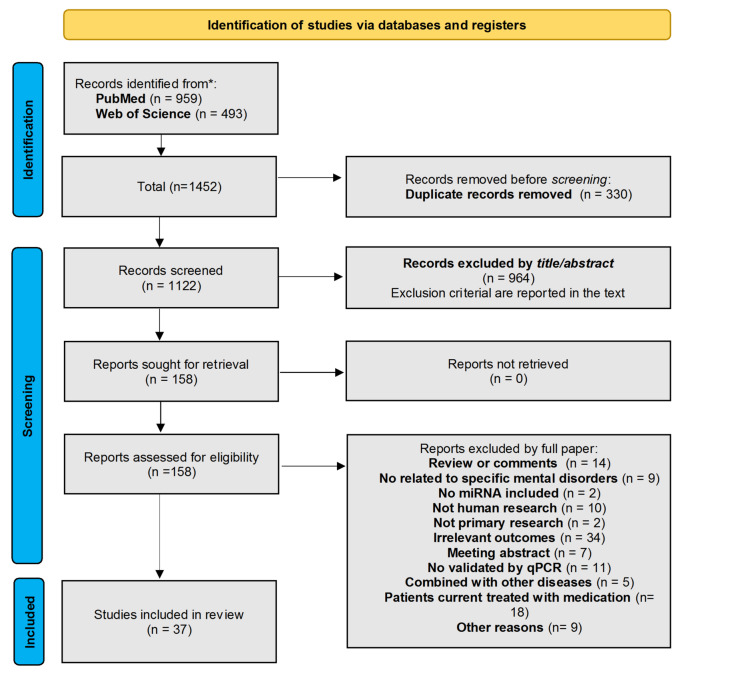
Flow diagram of study selection

According to the search criteria, initially, 1452 records were identified through the two databases (959 records from PubMed and 493 records from Web of Science); after removing 330 duplicate records, 1122 records were included for the screening by title and abstract, and 158 papers were left for the full-text analysis. Finally, 37 papers fulfilled the eligibility criteria, with a total of 2909 patients included in this systematic review (Fig. 1). Of them, 26 were case-control studies, and 11 were longitudinal studies that also followed a case-control study design but contained subsequent follow-up periods. For the aim of this systematic review, we focused only on the baseline miRNA alterations to exclude the potential confounding effects of treatment during the follow-up period. Therefore, regarding the alterations of miRNAs, both the case-control and the baseline data from longitudinal studies were included for analysis. Table 1 shows the characteristics of the studies included in this systematic review. Regarding the tissue source analyzed among the final included studies, 33 studies measured the miRNA expression levels in blood samples, mostly based on plasma and serum. Two studies measured miRNAs in brain tissues [22, 23], one study in cerebrospinal fluid (CSF) [24], and one study measured both in CSF and serum [25].

Regarding study quality assessment, the main limitations across the included studies were the lack of a specified and defined study population, no sample size justification, concurrent controls, blind outcome assessments, and confounding variables in the analysis. Three papers in particular had multiple of these limitations and did not have explicit inclusion and exclusion criteria for all participants or clearly defined cases/controls. Therefore, these three papers received the lowest score (a score of 3) and were considered having a high risk of bias [26–28] (see Supplementary File). Those papers were included in the results of this systematic review for completeness, but the reported results from those papers were excluded from the synthesis of the role of miRNAs and further discussion.

### The differential expressed MiRNAs in depressed people

In a comprehensive analysis of 37 studies comparing individuals with depression and control subjects, a total of 48 altered miRNAs were detected. Among those 48 altered miRNAs, after excluding data from studies with high bias, seven miRNAs exhibited high frequencies (≥ 3 occurrences) across different studies: miR-146a-5p (5 occurrences), miR-132-3p (4 occurrences), miR-124-3p (4 occurrences), miR-16-5p (4 occurrences), miR-155-5p (3 occurrences), miR-135a-5p (3 occurrences), and miR-451a (3 occurrences). For those miRNAs, two miRNAs exhibited consistent up-regulation in depressive patients compared with controls (miR-124-3p, miR-132-3p), and three miRNAs displayed consistent down-regulation in depressive patients (miR-135a-5p, miR-16-5p, miR-155-5p). Notably, two miRNAs showed inconsistent expression profiles across studies (miR-146a-5p, miR-451a). Characteristics of the studies included in this systematic review are summarized in Table 1.

**Table 1:** Characteristics of studies included in this systematic review

Author, year (Ref)	Research design	Diagnostic criteria	Sample size Case/control	Tissue	Methods	Total altered miRNAs	Up-regulated miRNAs	down-regulated miRNAs		NIH Quality Assessment score
Ahmadimanesh et al., 2023 [29]	Case-control (longitudinal)	DSM-IV	50/20	Blood (plasma)	qRT-PCR	3	miR-132-3p miR-124-3p		miR-16-5p	6/12
Aschrafi et al., 2016 [22]	Case-control	DSM-IV	5/8	Brain (Edinger–Westphal nucleus)	qRT-PCR	1			miR-326	5/12
Deng et al., 2022 [30]	Case-control (longitudinal)	DSM-IV	113/107	Blood (Serum EVs)	qRT-PCR	1	miR-146a-5p			7/12
Zhang et al., 2020 [31]	Case-control	DSM-V	50/30; Real-time PCR: 44/30	Blood (serum)	Real-time PCR	1	miR-9-5p			6/12
Brás et al., 2023 [32]	Case-control	DSM-V	32/40	Blood (PBMC)	RT-qPCR	3	miR-342-3p		miR-146a-5p miR-155-5p	5/12
Camkurt et al., 2015 [33]	Case-control	DSM-IV	50/41	Blood (plasma)	qRT-PCR	4	miR-451a miR-17-5p miR-223-3p		miR-320a-3p	5/12
Chen et al., 2020 [34]	Case-control	SCID-IV	32/18	Blood (plasma)	miRNA array, qRT-PCR	1			miR-19b-3p	8/12
Ding et al., 2021 [35]	Case-control	not mentioned	50/50	Blood (peripheral blood)	RT-qPCR	1			miR-135a-5p	5/12
Fan et al., 2014 [36]	Case-control	DSM-IV	81/46	Blood (PBMC)	miRNA array, qRT-PCR	5	miR-26b-5p miR-1972 miR-4485-3p miR-4498 miR-4743-5p			5/12
Fang et al., 2018 [37]	Case-control (longitudinal)	DSM-IV	45/32	Blood (plasma)	Real-time PCR	2	miR-132-3p miR-124-3p			4/12
Gheysarzadeh et al., 2018 [38]	Case-control	DSM-IV	39/36	Blood (serum)	RT-qPCR	3			miR-16-5p miR-135a-5p miR-1202	4/12
He et al., 2021 [39]	Case-control	DSM-V	40/34	Blood (peripheral blood)	Real-time PCR	1	miR-9-5p			6/12
He et al., 2016 [40]	Case-control (longitudinal)	SCID-IV	32/30	Blood (PBMC)	qRT-PCR	1	miR-124-3p			5/12
Hung et al., 2019 [41]	Case-control (longitudinal)	DSM-V	84/43	Blood (PBMC, monocytes)	qRT-PCR	4			let-7e-5p miR-21-5p miR-146a-5p miR-155-5p	7/12
Issler et al., 2014 [23]	Case-control	DSM-IV	*n* = 4–9 in RN and *n* = 6–11 in RM in each group	Brain (dorsal raphe (RN), raphe magnus (RM))	Real-time PCR	2			miR-135a-5p miR-16-5p	5/12
Kong et al., 2023 [42]	Case-control	DSM-V	52/52	Blood (plasma)	qRT-PCR	1	miR-26b-5p			6/12
Kuang et al., 2018 [43]	Case-control (longitudinal)	DSM-IV	84/78	Blood (serum)	qRT-PCR	3	miR-34a-5p miR-221-3p		miR-451a	6/12
Li et al., 2021 [44]	Case-control	DSM-IV-Text Revised	24/24	Blood (serum)	qRT-PCR	1			miR-27a-3p	5/12
Li et al., 2013 [45]	Case-control	Chinese classification of mental disorders	40/40	Blood (serum)	Real-time PCR	2	miR-132-3p miR-182-5p			5/12
Liu et al., 2021 [46]	Case-control (longitudinal)	DSM-IV	miRNA array: 5/5 qRT-PCT: 25/25	Blood (PBMC)	miRNA array qRT-PCR	2			miR-374b-5p miR-10a-5p	5/12
Lopez et al., 2014 [47]	Case-control	DSM-IV	32/18	Blood (peripheral blood)	qRT-PCR	1			miR-1202	4/12
Maffioletti et al., 2016 [48]	Case-control	DSM-IV-Text Revised	20/20	Blood (peripheral blood)	Microarray, Real-time PCR	7	miR-24-3p miR-425-3p miR-330-3p miR-345-5p		let-7a-5p let-7d-5p let-7f-5p	5/12
Marques et al., 2017 [49]	Case-control	DSM-IV	qRT-PCT: 8/9	Blood (plasma)	qRT-PCR	1			miR-19a-3p	5/12
Mendes-Silva et al., 2019 [50]	Case-control	DSM-V	NGS: 24/19 RT-qPCR: 39/34	Blood (plasma)	NGS, RT-qPCR	1			miR-184	5/12
Qi et al., 2018 [51]	Case-control	DSM-IV	81/123	Blood (peripheral blood)	qRT-PCR	1	miR-132-3p			5/12
Roy et al., 2017 [52]	Case-control	Mini International Neuropsychiatric Interview or the SCID-IV-Text Revised	18/17	Blood (serum)	qRT-PCR	1	miR-124-3p			5/12
Song et al., 2023 [26]	Case-control	DSM-IV	80/45	Blood (PBMC)	qRT-PCR	1	miR-4485-3p			3/12
Song et al., 2015 [24]	Case-control	DSM-IV	36/30	CSF	qRT-PCR	1			miR-16-5p	6/12
Sun et al., 2016 [53]	Case-control	DSM-IV and Chinese Version of the Modified SCID-IV-Text Revised	32/32	Blood (peripheral blood leukocytes)	qRT-PCR	2	miR-34b-5p miR-34c-5p			5/12
Tseng et al., 2023 [54]	Case-control (longitudinal)	DSM-V	30/28	Blood (PBMC)	qRT-PCR	2			miR-146a-5p miR-155-5p	6/12
Wan et al., 2015 [25]	Case-control	ICD-10 or DSM-IV	microRNA PCR Panel:6/6 qRT-PCR:32/21	CSF, blood (serum)	microRNA PCR Panel (CSF), qRT-PCR (serum)	4	let-7d-3p miR-34a-5p miR-221-3p		miR-451a	5/12
Wang et al., 2018 [27]	Case-control (longitudinal)	ICD-10 or DSM-IV	68/42, qRT-PCT:20/20	Blood (serum)	qRT-PCT	6	miR-132-3p miR-135b-5p miR-155-5p miR-181b-5p		miR-30e-5p miR-199b-5p	3/12
Wang et al., 2023 [55]	Case-control	DSM-IV	48/50	Blood (peripheral blood)	RT-qPCR	1			miR-16-2-3p	5/12
Wu et al., 2023 [56]	Case-control	not mentioned	24/24	Blood (serum, serum EVs)	qRT-PCR	1			miR-144-5p	5/12
Zhang et al., 2020 [57]	Case-control (longitudinal)	DSM-IV	35/35	Blood (plasma)	RT-qPCR	1			miR-134-5p	8/12
Hung et al., 2021 [58]	Case-control (longitudinal)	DSM-V	52/31	Blood (Serum EVs)	qRT-PCR	1	miR-146a-5p			7/12
Wei et al., 2020 [28]	Case-control	SCID-IV and ICD-10	qRT-PCR: 33/46	Blood (EVs)	genome-wide miRNA expression profiling, qRT-PCR	1	miR-139-5p			3/12

DSM: Diagnosis and Statistical Manual of Mental Disorders; SCID: Structured Clinical Interview for DSM; ICD: International Classification of Diseases; NGS: Next-generation sequencing; CSF: cerebrospinal fluid; EVs: extracellular vesicles; miRNA, miR: microRNA; PBMC: peripheral blood mononuclear cell; RT-qPCR: real-time quantitative polymerase chain reaction/quantitative reverse transcription polymerase chain reaction/reverse transcription and real-time quantitative polymerase chain reaction; qRT-PCR: quantitative real-time polymerase chain reaction; Real-time PCR: Real-time polymerase chain reaction

### MiRNAs associated roles regarding depression

**Fig. 2:**
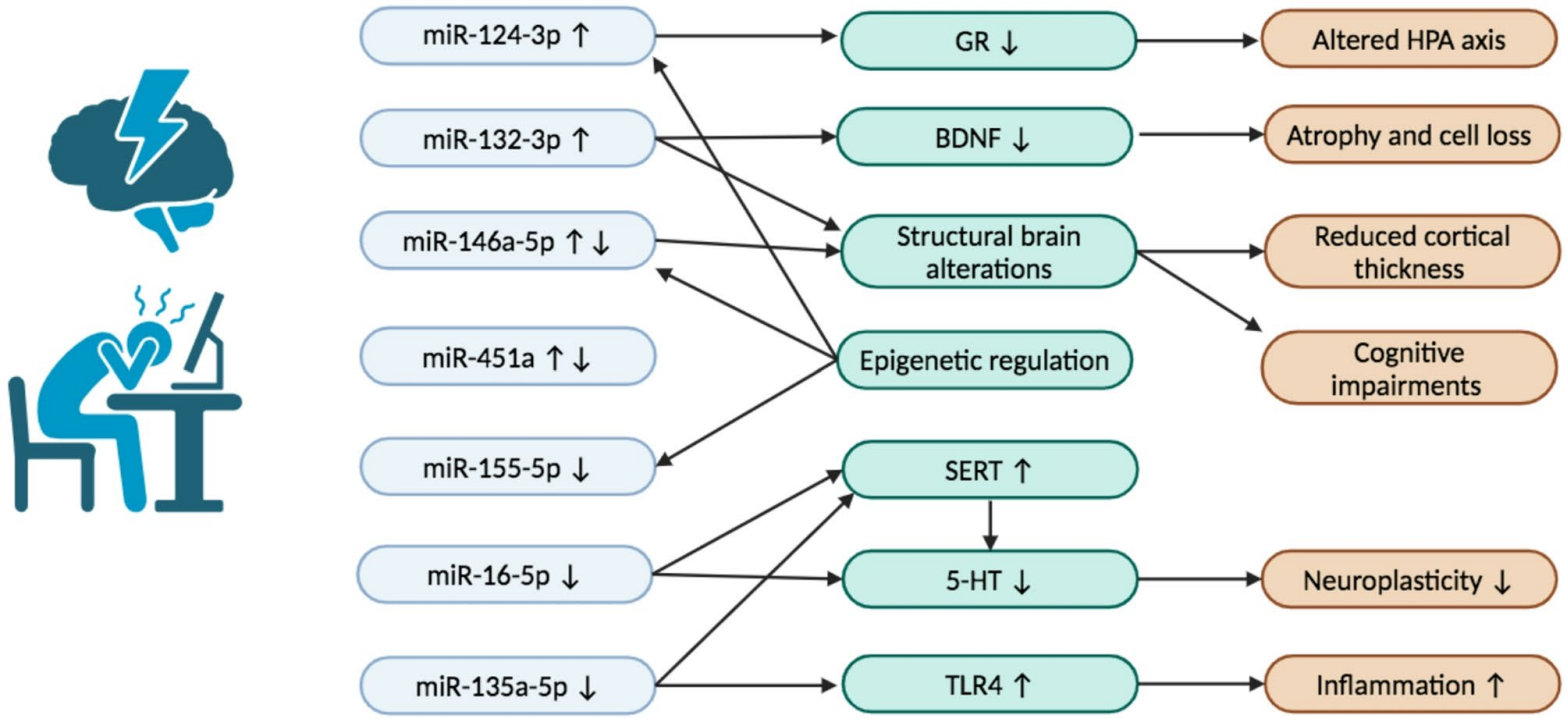
The frequently altered miRNAs in depression research and their associated roles in the pathophysiology of depression. ↑: up-regulated; ↓: down-regulated; miR: microRNA; GR: glucocorticoid receptor; BDNF: brain-derived neurotrophic factor; 5-HT: 5-Hydroxytryptamine, SERT: serotonin transporter/ 5-Hydroxytryptamine transporter; TLR4: toll-like receptor 4, HPA: hypothalamic-pituitary-adrenal

MiRNAs showed multiple roles regarding depression via different pathways, including miRNAs involved in neurobiological processes (miR-132-3p [45], miR-124-3p [29], miR-16-5p [24], and miR-135a-5p [35]), biomarkers for the diagnosis of depression (miR-16-5p [38], miR-135a-5p [38], and miR-451a [25]), miRNAs associated with depression symptoms/severity (HAMD, Self-Reporting Depression Scale; miR-146a-5p [41], miR-132-3p [37, 45], miR-16-5p [24], miR-155-5p [41], and miR-451a [43]), treatment outcomes (miR-146a-5p [58] and miR-124-3p [40]), epigenetic regulation (miR-124-3p [52], miR-146a-5p, and miR-155-5p [54]), and linked to structural brain changes (miR-146a-5p [30] and miR-132-3p [51]). The roles of those frequently occurring miRNAs are summarized in Table 2; Fig. 2.

**Table 2:** The roles of frequently occurring MiRNAs in the included studies

miRNAs	Category of role	Determined role in individual study
miR-146a-5p	Structural brain alterations	• The increased miR-146a-5p levels were associated with reduced cortical thickness [30].
	Epigenetic regulation	• The decreased H3K4me3 levels in the promoters of the genes encoding miR-146a-5p are involved in the psychopathology of depression [54].
	Depression severity	• miR-146a-5p was negatively correlated with HAMD-17 depression scores [41].
	Treatment marker	• miR-146a-5p levels showed predictability in achieving remission [58].
miR-132-3p	Influencing molecules involved in neurobiological processes	• Increased miR-132-3p levels is accompanied by a reduction in BDNF levels [29]. • A negative relationship between BDNF and miR-132-3p was found [45].
	Depression severity	• miR-132-3p was positive correlated with HAMD-17 scores [37]. • miR-132-3p was positive correlated with Self-Reporting Depression Scale [45].
	Structural brain alterations	• miR-132-3p associated with cognitive measures and symptoms [51].
miR-124-3p	Influencing molecules involved in neurobiological processes	• An increase in miR-124-3p levels is accompanied by a reduction in GR levels [29].
	Epigenetic regulation	• A set of miR-124-3p targeted genes were involved in stress response and neural plasticity [52]. • miR-124-3p is epigenetically regulated, and its interaction with the RNA-induced silencing complex is compromised in depression [52].
	Treatment marker	• miR-124-3p may be implicated in clinical response to antidepressant treatment in depressive patients [40].
miR-16-5p	Influencing molecules involved in neurobiological processes	• A decrease in miR-16-5p levels was associated with the increasing level of serotonin transporter [29]. • miR-16-5p was positively associated with CSF serotonin [24].
	Diagnosis marker	• miR-16-5p displayed a high sensitivity and specificity for the diagnosis of depression [38].
	Depression severity	• miR-16-5p was negatively correlated with HAMD-24 scores [24].
miR-155-5p	Epigenetic regulation	• The decreased H3K4me3 levels in the promoters of the genes encoding miR-155-5p are related to the psychopathology of depression [54].
	Depression severity	• miR-155-5p levels were positively associated correlated with HAMD-17 depression scores [41].
miR-135a-5p	Influencing molecules involved in neurobiological processes	• miR-135-5p regulated the apoptosis and inflammatory response via influencing the TLR4 expression [35]. • miR-135a-5p impacted 5-HT levels and metabolism [23].
	Diagnosis marker	• miR-135a-5p displayed a high sensitivity and specificity for the diagnosis of depression [38].
miR-451a	Depression severity	• miR-451a level was negative correlated with HAMD-24 scores [43].
	Treatment marker	• miR-451a level was positively associated with antidepressant efficacy [43]. • miR-451a displayed a high sensitivity and specificity for diagnosis of depression [25].

↑: up-regulated; ↓: down-regulated; miRNA, miR: microRNA; HAMD: Hamilton Depression Rating Scale; CSF: cerebrospinal fluid; GR: glucocorticoid receptor; BDNF: brain-derived neurotrophic factor; 5-HT: 5-Hydroxytryptamine

## Discussion

The main goal of this study was to systematically review the current literature regarding altered miRNA expressions in the pathophysiology of depression. We identified 48 altered miRNAs from 37 depression studies. Many of these 48 observed and altered miRNAs were only researched once or twice among the included studies and lacked enough evidence to prove their role in depression. In order to provide a more reliable understanding and prevent misinterpretation of the effect of miRNAs involved in depression, we concentrated on the seven miRNAs that appeared most frequently across different depression studies, including miR-146a-5p, miR-132-3p, miR-124-3p, miR-16-5p, miR-155-5p, miR-135a-5p, and miR-451a. Clarifying the complex landscape of miRNA involvement in depression to its most impactful components provides us with a clearer insight into the potential role of miRNAs in depression. For instance, of them, several altered miRNAs (miR-146a-5p, miR-132-3p, miR-16-5p, miR-155-5p, and miR-451a) showed associations with depression severity, measured by the Hamilton depression rating scale (HAMD) [59], which revealed the potential of miRNAs in assessing and monitoring the severity of depression. Further, some miRNAs showed their clinical relevance in multiple aspects, including predicting achieving remission (miR-146a-5p [58]), responding to antidepressant treatment (miR-124-3p [40], miR-451a [43]), and diagnosis of depression (miR-16-5p, miR-135a-5p [38], and miR-451a [25]), all these results emphasize the promise of the clinical application of miRNAs.

As the most frequently involved miRNA in the included studies, miR-146a-5p was found to be associated with structural brain alterations, epigenetic regulation, and clinical roles. Increased miR-146a-5p levels were related to the reduced cortical thickness in several brain sites, indicating its potential impact on structural brain changes associated with depression [30]. Additionally, the levels of miR-146a-5p were epigenetically regulated via histone modification [54], which contributes to the psychopathology of depression. Besides the here included studies, other researchers have highlighted the effects of miR-146a-5p in immune systems, which share many common characteristics with nervous systems. The change of multiple inflammatory molecules has been reported in depressed patients, for example, pro-inflammatory cytokines and chemokines, including tumor necrosis factor-α, interleukin (IL)-1β, IL-2, IL-6, IL-12, and C-reactive protein, were found increased in the blood from patients with depression. Still, the anti-inflammatory cytokines, such as IL-4 and IL-18, were reduced in depressed patients [60]. Stress-based alterations in the immunity and inflammation in peripheral organs, orchestrated by the HPA axis, as well as sympathetic and parasympathetic nervous systems, affect the interaction between neurons and glial, resulting in neuroinflammation and behavior phenotypes, which underlay the neurobehavioral outcomes related to depression [60]. MiR-146a-5p, an anti-inflammatory miRNA, was reported as a mediator in the neuroimmune system homeostasis and affects the pathogenesis of neuroinflammatory disorders [61]. For instance, miR-146a-5p limits the infiltration of inflammatory cells, declining the release of inflammation-related mediators, thus mitigating neuroinflammation [61], which emphasizes the role of miR-146a-5p in neuroinflammatory disorders, such as depression. Notably, the expression profiles of miR-146a-5p were inconsistent across studies, with the up-regulated miR-146a-5p all originating from serum EVs, while the downregulated miR-146a-5p were all originating from PBMCs and monocytes. Since miRNAs are variably expressed in different blood cells [62], the inconsistent alterations of miR-146a-5p may be attributed to the different origins.

Several miRNAs, including miR-132-3p, miR-124-3p, miR-16-5p, and miR-135a-5p, impact the molecules involved in neurobiological processes and thus might play a role in the pathogenesis of depression. For example, brain-derived neurotrophic factor (BDNF), a secretory protein in the neurotrophic family, controls a series of spectrums involved in neuroplasticity processes [63]. Previous studies support “neurotrophic hypothesis of depression”, which suggests that reduced BDNF levels in the brain lead to atrophy and cell loss in depression, while antidepressants exert their therapeutic effects via increasing BDNF levels [64]. Several included studies came to the same result, demonstrating that increased miR-132-3p was associated with decreased BDNF levels [29, 45], suggesting the role of miR-132-3p in neuroplasticity alterations. Moreover, miR-132-3p was related to brain function and structure, and its altered levels result in the loss of specific brain areas and associated cognitive impairments [51], which empathize the role of miR-132-3p in the pathogenesis of depression.

As another consistent up-regulated miRNA, different studies showed that miR-124-3p targeted genes were associated with several essential biological pathways related to depression, including stress response and neural plasticity [40, 52]. MiR-124-3p was epigenetically regulated, which revealed the interaction between genes and the environment [52]. Hyperactive HPA axis and subsequently increased levels of cortisol levels were shown in depressive patients, and the effects of cortisol were mediated by the activating of glucocorticoid receptor (GR), which is also regarded as a regulator for several neurotrophic factors such as BDNF [65]. Ahmadimanesh et al. [29] found that reduced GR levels were inversely related to the increased miR-124-3p levels in depressive patients, suggesting the potential role of miR-124-3p in the altered activity of HPA axis and the progress of depression.

Serotonin neurotransmitters activate the 5-HT receptors; the serotonin neurotransmitter can be taken up by 5-HT transporters (SERT) from the synaptic cleft. Both the 5-HT and SERT have an essential role in depression [66]; for instance, 5-HT was regarded as a vital neuromodulatory transmitter with specific neuroplastic properties [67], and previous research revealed that depressive patients accompanied with lower levels of 5-HT [68], which is a risk factor of depression. The effect of classical antidepressants (selective serotonin reuptake inhibitors) was to reduce the SERT, then inhibit the reuptake of 5-HT and act their pharmacological effects [69]. Therefore, considering the finding of miR-16-5p from included studies, its potential role can be understood as decreased miR-16-5p levels associated with increased SERT [29] and decreased 5-HT levels [24], contributing to the progress of depression via involvement in neuroplastic properties.

Similar to miR-146a-5p, miR-155-5p was also epigenetically regulated via histone modification [54], histone 3 lysine 4 tri-methylation, which may results in the altered expression of downstream messenger RNA in depressive patients, thus related to the psychopathology of depression. Moreover, depressive patients showed reduced levels of histone 3 lysine 4 tri-methylation in the promoter region of toll-like receptor 4 (TLR4), a well-known protein in mediating the inflammatory response to stress [70], showed the potential role of epigenetic modification in influencing the inflammatory processes in depressive patients [54]. A decreased level of miR-135a-5p in depressive patients was observed in included studies; amongst them, Ding et al. [35] found that miR-135a-5p inhibits its targeted gene, TLR4, in the hippocampus and may protect against depression in animal models. Thus, the decreased miR-135a-5p levels in depressive patients may concurrently decrease its repressive role on TLR4, leading to stronger inflammatory effects in corresponding tissues, such as the brain, and enhancing susceptibility to depression. Furthermore, Issler et al. [23] found increased miRNAs of miR-135a-5p after antidepressant treatment, which is associated with its effect on the alteration of tissue 5-HT levels and metabolism; therefore, miR-135a-5p was regarded as an endogenous antidepressant, further explains the reduced levels of miR-135a-5p in depressive patients.

Regarding miR-451a, the included studies did not address its specific mechanisms in depression, focusing only on its association with depression severity [43] and some clinical relevance [25, 43]. Other research demonstrated a decreased level of miR-451a due to ketamine (antidepressant), showing its potential role during antidepressant treatment [71]. Current findings indicate that miR-451a targets activating transcription factor 2 and inhibits the transcription of corticotropin-releasing hormone receptor 1, potentially impacting the neuronal structural plasticity and maintaining homeostasis of the HPA axis in response to stress [72]. Interestingly, miR-451a exhibits different expression patterns across included studies (increased in plasma and decreased in serum from depressed patients), considering that these studies regarding miR-451a took similar experimental approaches (qRT-PCR), same diagnostic criteria (DSM-IV), and current experimental demonstration that there is no significant difference of miR-451a levels between serum and plasma [73], which remains to be explained by more studies.

### Strengths and limitations

As the most common type of mental disorder, the considerable social impact and substantial burden of depression worldwide contribute to the high research interest in depression within the field of miRNAs. However, the presence of a high risk of bias in these studies requires a cautious interpretation of these results. Although strict eligibility criteria were set to control the factors that may affect the level of miRNAs, such as medications and experimental methods, there are still some factors that may affect their expression, such as inconsistent diagnostic criteria for depression and different tissue sources of miRNAs. Also, meta-analysis cannot be implemented because of the heterogeneity across studies; for example, there are different criteria for diagnosis and severity of depression, as well as incomplete data reporting, as many studies only report the dysregulated expression levels of miRNAs instead of the exact miRNA expression levels.

These findings underlie the potential of miRNAs as vital regulatory molecular factors in the pathophysiology of depression. However, the inconsistency of miRNA expression profiles in all included studies suggests the need for standardization in multiple aspects, including sample processing, diagnosis criteria, patient selection, and tissue from which the miRNA was derived. Additionally, the sample sizes of most studies range from 20 to 80, so future studies should focus on larger cohorts to confirm or deny those mechanisms regarding depression we synthesized. The interaction between miRNAs and other molecular mechanisms, such as the gut-brain axis and mitochondrial allostatic load, should be further considered, which could provide a more comprehensive understanding of their role in depression.

## Conclusion

This systematic review revealed the altered expression of miRNAs across multiple depression studies, and the synthesized role of highly frequent miRNAs with altered expressions were enriched in influencing molecules involved in neurobiological processes, such as 5-HT, SERT, and BDNF. This work contributes to the relevant field by reviewing the role of miRNAs as possible biomarkers for the diagnosis, therapeutic targets, and treatment response regarding depression. Integrating miRNA analysis into clinical practice notes the promise for personalized medical approaches in effective diagnostic and therapeutic strategies.

## Electronic supplementary material

Below is the link to the electronic supplementary material.

Supplementary Material 1

## Data Availability

Data supporting the findings of this study are available from the corresponding author upon reasonable request.

## Abbreviations

5-HT: 5-Hydroxytryptamine
BDNF: Brain-derived neurotrophic factor
CSF: Cerebrospinal fluid
DSM: Diagnosis and Statistical Manual of Mental Disorders
EVs: Extracellular vesicles
GR: Glucocorticoid receptor
HAMD: Hamilton depression rating scale
HPA: Hypothalamic-pituitary-adrenal
hsa: *Homo sapiens*
ICD: International Classification of Diseases
IL: Interleukin
miRNA, miR: microRNA
NGS: Next-generation sequencing
PBMC: Peripheral blood mononuclear cell
PRISMA: Preferred reporting items for systematic reviews and meta-analyses
qRT-PCR: Quantitative real-time polymerase chain reaction
RT-qPCR: Real-time quantitative polymerase chain reaction/quantitative reverse transcription polymerase chain reaction/reverse transcription and real-time quantitative polymerase chain reaction
RT-PCR: Real-time polymerase chain reaction
SCID: Structured Clinical Interview for DSM
SERT: Serotonin transporter/5-Hydroxytryptamine transporter
TLR4: Toll-like receptor 4
